# Supplementary material for: Mortality and associated risk factors of COVID-19 infection in dialysis patients in Qatar: A nationwide cohort study
Source: PLoS One. 2021 Jul 22;16(7):e0254246. doi: 10.1371/journal.pone.0254246 (PMC8297751; doi:10.1371/journal.pone.0254246)
Supplement: Attachment — Submitted filename: Response to Reviewers PLOS1.docx [file pone.0254246.s002.docx]

Dear Editor in Chief and respected reviewers:

We like to thank the reviewers for their valuable comments. We appreciate all your efforts. We feel that your comments added a lot of value to our manuscript and we tried to answer all of them as suggested.

1. TITLE
Please include mortality in the title since the paper is focused in associated death risk.

title modified as recommended to:

**Mortality and associated risk factors of COVID-19 infection in dialysis patients in Qatar: a nationwide cohort study**

2. ABSTRACT
Methods: Recommended to emphasize these patients were on maintenance dialysis therapy.

Added to the method section of the abstract

Please mention univariate regression analysis in this section since line 97 is a result of this test.
Done and added to methods section

Results: Line 89: Do the authors refer as patients under dialysis without covid-19 as general population?

Thank you for this valuable observation and comment. We corrected this part and removed the comparison (both in incidence and mortality) to the general population of Qatar from the result section. Instead we felt it would be useful to add some national data for general comparison in State of Qatar (we added reference (8) regarding COVID-19 in Qatar) in the discussion section.

3. INTRODUCTION
Line 129: Does general population mean patients under dialysis? The abstract is using “general population” as control, thus, it is understood that those patients were also under dialysis.
We appreciate your observation. We meant by general population as all population of the country (national data). We removed the term general population from the abstract and the result section to clarify that. We added small reference to data from national general population in Qatar in the discussion section to highlight difference to our cohort (dialysis patients).

As suggested later, we added control group in table 1 (COVID-19 negative dialysis patients).

Line 141: Recommended to not include objectives in this section since this overlap with the primary and secondary outcomes mentioned in the methods section. Recommended to substitute with “The purpose of this study is to determine the incidence and mortality of covid-19 infection in patients under dialysis in the state of Qatar".

Changes done as advised.

4. METHODS
Please describe the exclusion criteria from Figure 2 in this section.

Both inclusion and exclusion criteria added to the methods section

The primary outcome (mortality) is not clear. Recommended to include the biostatistical equation for mortality.

Done and added to methods

Line 206: What about predictors for incidence?
Did the authors performed a regression analysis adjusted to potential confounders?

Thank you for your suggestion. We modified (Table1) to include control group (dialysis without COVID19 and highlighted it in the results. We performed comparison of our dialysis patients with COVID19 (study cohort) and without COVID19 (control) regarding main demographic, comorbidities etc. We included differences in secondary objective 1.

5. RESULTS
Line 212: The abstract mentions 56 patients with HD, and 20 with PD. Please revise.

line 212 is correct but line 88: revised in the abstract: 65 hemodialysis and 11 peritoneal dialysis

Lines 214, 215, 216: Please include denominators for sample size (n/N, %).

All denominators/% added as recommended.

Primary outcome:

Please utilize denominators in this paragraph as well.

done as recommended

Again, line 231 refers to general population in Qatar regardless of dialysis.

Thank you for comment, we removed this part and mentioned it in part in the discussion as general information about general population in Qatar.

We added the comparison group of dialysis patient not infected with COVID19 in Table 1

Recommended to report significant associations with mortality from table 2 as text.

We reported significant associations for mortality as recommended.

Line 241-244: Report p-value and 95% CI next to OR.

Done in text as recommended

Secondary outcomes: Line 257-260 please elaborate.

Agree with comment. We removed this part regarding general population of Qatar and mentioned it in part in the discussion as general information about general population in Qatar.

Line 262: include objective data for total dialysis population?.
Recommended to include association of risk factors with incidence of covid-19 in patients with dialysis.

Appreciate the comment. We added the recommended objective data from general dialysis patients (Table1) and highlighted it in the results. We performed comparison of our dialysis patients with COVID19 (study cohort) and without COVID19 (control) regarding potential risk factors of incidence like demographics, comorbidities etc.

Severity and complications: Please include denominators (n/N) next to %

Added as recommended

What was the mean ICU length of stay in those 19 patients admitted?

Done in details as recommended

Comparison of PD vs HD: line 287, 289: it cannot be claimed that incidence is lower since difference was not significant.

corrected text as recommended

Line 292: What do the authors mean by comparable?

Thank you for this observation. We meant not with statistically significant different, we clarified in text.

Line 292, 293, 295 and 296: Please include objective data.
done as recommended in the text.

6. DISCUSSION
Line 312-316 should be moved to the introduction section.

done as recommended

321: general population with or without dialysis?

As explained above, we mentioned difference between incidence and mortality of COVID19 in dialysis and in general population in Qatar (from available Data Ref 8) to highlight the difference.

331: Please substitute “ten times greater” by “showed a trend of” since p value was not significant.

Agree with your comment, we modified as recommended

335: Please describe the socioeconomic status from reference 13.

We agree with this valuable comment. After careful review and further literature search, we opted to exchange reference 13 with 2 updated references from different population as below and added to text.

Oh et al. found that lower socioeconomic status was associated with a higher risk of contracting COVID-19 especially in the older population while Hawkins et al. found that lower education levels, median income and poverty rate were strongly associated with higher rate COVID-19 cases.

14- Tak Kyu Oh, Jae-Wook Choi, In-Ae Song: Socioeconomic disparity and the risk of contracting COVID-19 in South Korea: an NHIS-COVID-19 database cohort study. BMC Public Health (2021) 21:144

# 15- R. B. Hawkins, E. J. Charles, J. H. Mehaffey: Socio-economic status and COVID-19–related cases and fatalities. *Public Health*, Volume 189, December 2020, Pages 129-134

# 348: Please include the x-ray previous reports from reference 18.

As you recommended, we added details to text:

This is similar to previous report by Vancheri et al. who described that among 240 patients with COVID19 who underwent chest X ray, 73.3% showed bilateral lung alteration (infiltrates, reticular or ground glass opacity) with only 25% showed negative X ray.

352: Peak levels for blood workout in HD vs baseline levels in PD is not a fair comparison. In addition, it is not recommended to state “higher peaks” if difference was not significant.

You are right and thank for this observation. We had a typo here. We clarified it in the text. It does match the results now. We differentiated between peak and base significant differences between PD and HD.

355: DVT and AF were significantly associated with mortality in chi2 test, but regression analysis was not performed nor reported. Therefore, if the authors use chi2 to report association with mortality, then all chi2 values should also be included. This means chest x ray, and all significant laboratory findings. Recommended to perform regression analysis for all variables.

Thank you for your valuable comment, univariate Cox regression analysis was redone for all variables as requested and results section adjusted accordingly. Table 2 was separated to tables (2 and 2A) to clarify data analysis. Table 2 shows comparison of survivors to non-survivors with COVID19 using T-test for continuous variables and Chi-square statistical analysis for categorical variables. Table 2A shows odd ratios for risk factors in non-survival group using univariate Cox regression analysis.

357: The only significant differences of peak levels for non-survivors were CRP, ferritin, il-6, and WBC count, the rest of the inflammatory markers were not higher. In addition, peak levels for CK, WBC, D-dimer, liver enzymes were not reported. Where is this information coming from? Please revise.

Thank you for your comment. Data revised and changed as advised.

363: Case fatality rate should be reported in the results section. This data was not described before.
Thank you for pointing this out. Sorry, this term was used inappropriately. We changed it in text to death rate as clarified in the methods too.

7. TABLES
Table 1: The article compares patients under dialysis with and without covid-19 throughout the manuscript (incidence). It is recommended to compare both populations in table 1, including baseline laboratory findings (bloodwork).

Per your valuable feedback, we added (Table1) and highlighted it in the result. We performed comparison of our dialysis patients with COVID19 (study cohort) and without COVID19 (control) regarding main demographic, comorbidities etc. Baseline laboratory cannot be compared as most values are not done routinely in general dialysis population. But we added graph as recommended to compare baseline labs upon diagnosis versus day7 (as we had only 2 values significantly changed between baseline and day 7(lymphocyte count and albumin), we opted to add also values with significant change between baseline and peak too (4 variables were significant).

Table 2 (Mortality): Recommended to describe those significant associations in regression analysis in the Results section.

Agree with comment and revised as recommended.

Please be specific for regression model or chi2. This creates confusion.

We agree with the comment. We separated table 2 to table 2 and 2A as above to clarify this point and fulfill request to perform complete regression analysis for all variables.

Table 3: Recommended to remove table 3 and report complications as text in the Results section.

Done as recommended.

Moreover, it is recommended to illustrate in a figure those laboratory findings that were significantly different (t-test) between baseline and 7 days.

Done as above. We added the graph as recommended to compare baseline labs upon diagnosis versus day7 (as we had only 2 values significantly changed between baseline and day 7 (lymphocyte count and albumin), we opted to add also values with significant change between baseline and peak too (4 values were significant).

8. FIGURES
Figure 1. Please use a standard flow chart for retrospective studies.

Done as recommended and attached in the manuscript

Recommended to remove inclusion and exclusion criteria (this should be explained as text).

removed and added to methods.

Done and added to methods

Recommended to include the number (n=971) of the first filter of excluded patients with reason of exclusion. Same for those excluded after “assessed for eligibility”.

Done as recommended

those significant associations with mortality and incidence (after revision) in regression analysis should be further discussed in the correspondent section.

done as recommended

2. Thank you for stating in the text of your manuscript "the study meets the waiver criteria of informed consent, and all data were fully anonymized before we accessed them". Please also add this information to your ethics statement in the online submission form.

done as recommended

3. Thank you for providing the date(s) when patient medical information was initially recorded. Please also include the date(s) on which your research team accessed the databases/records to obtain the retrospective data used in your study."

Thank you for your comment. We mentioned this in the study methodology section:

Data has been accessed from 25 July- 25 October 2020.

4. Please include captions for your Supporting Information files at the end of your manuscript, and update any in-text citations to match accordingly. Please see our Supporting Information guidelines for more information: [http://journals.plos.org/plosone/s/supporting-information](about:blank).

Done as recommended

While revising your submission, please upload your figure files to the Preflight Analysis and Conversion Engine (PACE) digital diagnostic tool, <https://pacev2.apexcovantage.com/>. PACE helps ensure that figures meet PLOS requirements. To use PACE, you must first register as a user. Registration is free. Then, login and navigate to the UPLOAD tab, where you will find detailed instructions on how to use the tool. If you encounter any issues or have any questions when using PACE, please email PLOS at [figures@plos.org](mailto:figures@plos.org). Please note that Supporting Information files do not need this step.

Done as recommended.
