# Supplementary material for: Mortality and associated risk factors of COVID-19 infection in dialysis patients in Qatar: A nationwide cohort study
Source: PLoS One. 2021 Jul 22;16(7):e0254246. doi: 10.1371/journal.pone.0254246 (PMC8297751; doi:10.1371/journal.pone.0254246)
Supplement: Attachment — Submitted filename: 2nd response to reviewer.docx [file pone.0254246.s003.docx]

PONE-D-21-00180R1
Mortality and associated risk factors of COVID-19 infection in dialysis patients in Qatar: a nationwide cohort study
PLOS ONE

Dear Editor in Chief and Esteemed Reviewers:

Thank you for your valuable review and feedback

please find our rebuttal letter that responds to each point raised 'Response to Reviewers'.

I want to highlight that we had help from a statistician this time (we added acknowledgment) to fulfill the valuable recommendations of reviewers.

Journal Requirements:

Please review your reference list to ensure that it is complete and correct.

We reviewed and updated all references as per journal guidelines. We checked and found no reference has been retracted

I would recommend to check for inconsistent fonts within the manuscript.

thank you for your valuable comment. We made changes necessary as recommended.

Abstract

The abstract should follow a proper sequence. Title says “mortality and associated risks”, but the context says “incidence and outcomes”; these are not the same.

Thank you for this valued observation. We agree with comment. text changed as recommended.

Methods is not describing the primary (mortality and associated risk factors [page 19]) and secondary (a, b, and c) outcomes; this creates confusion on what the results are describing.

Agree with remark. Primary and secondary objectives added to methods

In the results: “Male patients had double risk for contacting COVID-19”. This is an associated risk for incidence, not mortality. Do the authors mean risk for death?

Thank you for this comment. We rephrased to double the incidence to make it clear and not related to mortality and avoid any confusion.

The authors should include the associated risk factors for mortality described in page 19/table 2A.

Agree with your valuable observation. We added the most important significant risk factors from Table 2A (now 3A) as advised. Also added result from new table 3B (multivariate Cox regression analysis).

The conclusion should state “higher incidence compared to the general population of Qatar” since the 7.1% is higher than prior reports (4%). Same for mortality (15% vs 0.15%). Recommended to clarify these “high” numbers are compared to nationwide.

Thank you for your comment. We added and rephrased the conclusion in the abstract as suggested.

Body of manuscript
Statistical analysis

The survivor vs non-survivor cohorts have vast significant differences/trends for baseline values (Page 18 and 19/Table 2). Did the cox regression analysis include those as potential confounders for associated risk factors for mortality? Did the authors perform a selection method (i.e., forward, backward, stepwise) to determine truly potential confounders from table 2?
Thank you for your valuable comments. The cox regression analysis summarized in table 2A (now changed to 3A) was a univariate analysis and did not account for potential cofounders. Therefore, we constructed in this revision a forward stepwise multivariate cox regression analysis model to further examine the association between the different statistically significant variables in the univariate analysis (Table 2A, p < 0.05) and mortality in dialysis patients infected with COVID-19. The model initially included clinical variables that were statistically significant (p<0.05) and that were thought to be clinically relevant and not collinear. ICU admission was found to be the only clinical variable significantly associated with death [OR = 32.8 (3.5-305.4), p=0.002)] (Table 3B). Then we tried to add the blood investigation variables that were statistically significant in the univariate analysis (Table 2A, p<0.05) to the model. However, the model became unstable due to our small sample size as well as the collinearity between ICU admission and inflammatory and thrombotic blood markers such as lactate, CRP, IL-6, D-dimer and ferritin.

Results

Denominator in line 221 and the flow chart is 1064. The abstract and other sections have 1068, please revise.

Agree and we commend you for sharp observation and we apologize for the typo. all corrected to the correct number 1064.

For secondary outcome C (line 380), recommended to describe only significant values within the text to avoid confusion.

Thank you for the comment. We summarized (very brief) non-significant difference in one sentence and kept the significant ones as advised. For incidence and mortality, we kept it to highlight these important factors though it was not statistically significant.

Discussion

Line 468: Recommended to avoid using “some association”. Whether there is an association or not.

Agree with comment. We rephrased to association with multiple inflammatory markers

470: “Mortality was more associated with older age and dialysis”. What do the authors mean as “more”. Again, associations should be determined or not. Are these results described previously?
Appreciate this observation. We removed more as advised.

474: Recommended to report the percentages for case fatality rate of Spain, Italy, and Turkey.
Done as recommended (as mortality rate in Turkey study was lower than ours, we refrased the sentence to lower than most international figures.

Figure 1: I believe there is a typo on the screening box. “tested negative” should be “positive”??
Yes and again thank you for your close observation and sorry for the typo. Changes to positive.

Figure 3: Recommended to use panel labels for each graph (A, B, C, etc).

Graph labeled as recommended.

Error bars for albumin and lymphocytes show non-significant difference. Please revise

Agree with valuable comment. After consulting with our statistician and reanalysis, we adjusted the graph
